# Rauniticine-*allo*-oxindole B methanol monosolvate

**DOI:** 10.1107/S1600536811016710

**Published:** 2011-05-07

**Authors:** Fatimah Salim, Rohaya Ahmad, Nor Hadiani Ismail, Hazrina Hazni, Seik Weng Ng

**Affiliations:** a Faculty of Applied Sciences, Universiti Teknologi MARA, 40450 Shah Alam, Selangor, Malaysia; b Department of Chemistry, University of Malaya, 50603 Kuala Lumpur, Malaysia

## Abstract

The title penta­cyclic oxindole alkadoid, isolated from *Uncaria longiflora*, crystallizes as a methanol solvate, C_20_H_22_N_2_O_4_·CH_4_O. The five-membered ring comprising the indole fused ring is nearly planar [maximum atomic deviation = 0.031 (2) Å], whereas the five-membered ring having alphatic C atoms adopts an envelope shape (with the tertiary N atom representing the flap). The six-membered ring that shares an N atom with the envelope-shaped ring adopts a chair shape; the six-membered ring having an O atom is sofa-shaped. The carb­oxy­lic acid group acts as a hydrogen-bond donor to a methanol mol­ecule; this, in turn, acts as a hydrogen-bond donor to the double-bond carboxyl O atom of an adjacent mol­ecule, generating a chain. Adjacent chains are linked by N—H⋯O hydrogen bonds, forming a layer motif.

## Related literature

For the spectroscopic identification of rauniticine-*allo*-oxindole B, see: Seki *et al.* (1993 ▶).
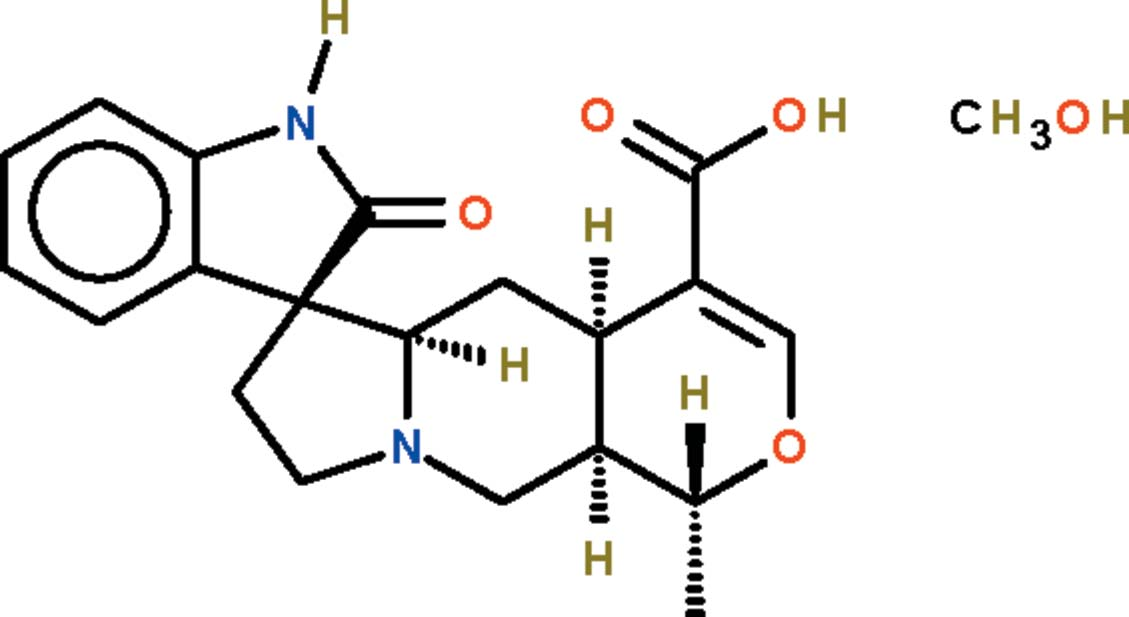


## Experimental

### 

#### Crystal data

C_20_H_22_N_2_O_4_·CH_4_O
*M* *_r_* = 386.44
Monoclinic, 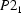

*a* = 9.2330 (3) Å
*b* = 7.2110 (2) Å
*c* = 14.7678 (4) Å
β = 99.313 (3)°
*V* = 970.27 (5) Å^3^
*Z* = 2
Mo *K*α radiation
μ = 0.10 mm^−1^
*T* = 100 K
0.20 × 0.10 × 0.05 mm

#### Data collection

Agilent SuperNova Dual diffractometer with an Atlas detector
Absorption correction: multi-scan (*CrysAlis PRO*; Agilent, 2010 ▶) *T*_min_ = 0.981, *T*_max_ = 0.995
9109 measured reflections
2381 independent reflections
2181 reflections with *I* > 2σ(*I*)
*R*_int_ = 0.039

#### Refinement

*R*[*F* ^2^ > 2σ(*F* ^2^)] = 0.037
*wR*(*F* ^2^) = 0.093
*S* = 1.05
2381 reflections
266 parameters
4 restraints
H atoms treated by a mixture of independent and constrained refinement
Δρ_max_ = 0.21 e Å^−3^
Δρ_min_ = −0.19 e Å^−3^

### 

Data collection: *CrysAlis PRO* (Agilent, 2010 ▶); cell refinement: *CrysAlis PRO*; data reduction: *CrysAlis PRO*; program(s) used to solve structure: *SHELXS97* (Sheldrick, 2008 ▶); program(s) used to refine structure: *SHELXL97* (Sheldrick, 2008 ▶); molecular graphics: *X-SEED* (Barbour, 2001 ▶); software used to prepare material for publication: *publCIF* (Westrip, 2010 ▶).

## Supplementary Material

Crystal structure: contains datablocks global, I. DOI: 10.1107/S1600536811016710/xu5205sup1.cif

Structure factors: contains datablocks I. DOI: 10.1107/S1600536811016710/xu5205Isup2.hkl

Additional supplementary materials:  crystallographic information; 3D view; checkCIF report

## Figures and Tables

**Table 1:** Hydrogen-bond geometry (Å, °)

*D*—H⋯*A*	*D*—H	H⋯*A*	*D*⋯*A*	*D*—H⋯*A*
O1—H1⋯O5	0.84 (3)	1.83 (3)	2.662 (3)	173 (4)
O5—H5⋯O2^i^	0.84 (3)	1.91 (3)	2.728 (2)	165 (4)
N2—H2⋯O4^ii^	0.88 (3)	1.97 (3)	2.805 (3)	158 (3)

Symmetry codes: (i) 
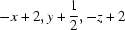
; (ii) 
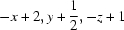
.

**Fig. 1.**
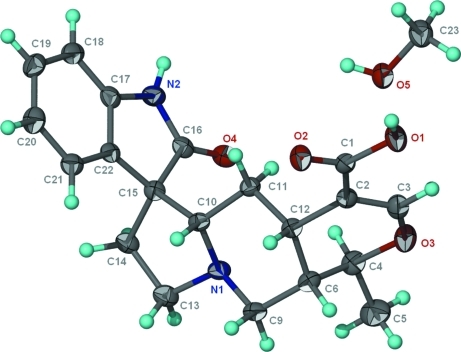
Thermal ellipsoid plot (Barbour, 2001) of C20H22N2O4.CH3OH at the 70% probability level; hydrogen atoms are drawn as spheres of arbitrary radius.

**Fig. 2.**
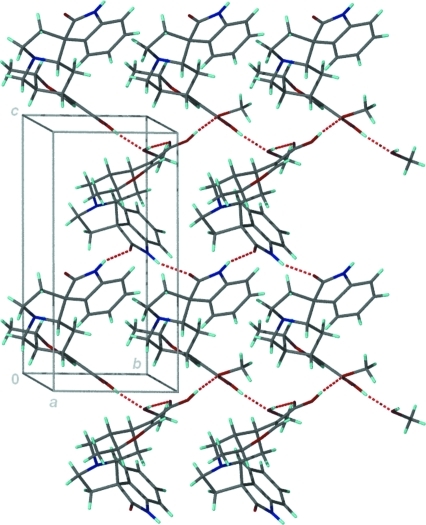
Hydrogen-bonded layer structure.

## supplementary crystallographic information

### Comment

The genus *Uncaria* is a source of diverse bioactive compounds, and parts of the plant are use for medicinal purposes. The structure of rauniticine-*allo*-oxindole B was previously elucidated by NMR spectroscopy in on study on heteroyohimbine-type oxindole alkaloids (Seki *et al.*, 1993). The assignment is confirmed in the present study on the methanol-solvated compound (Scheme I) isolated from *Uncaria longiflora*. The pentacyclic oxindole alkadoid, C_20_H_22_N_2_O_4_, features a five-membered ring that adopts the shape of an envelope (with the tertiary N atom representing the flap). The six-membered ring that shares an N atom with the envelope-shaped ring adopts the shape of a chair; the six-membered ring having an O atom is sofa-shaped (Fig. 1). The carboxylic acid portion of the molecule is hydrogen-bond donor to a methanol molecule; this, in turn, is hydrogen-bond donor to the double-bond carboxyl O atom of an adjacent molecule to generate a chain. Adjacent chains are linked by an N–H···O hydrogen bond to form a layer motif (Table 1, Fig. 2).

### Experimental

*Uncaria longiflora* plant material was collected from Hutan Simpan Bangi, Selangor, Malaysia, and specimens were deposited at Taman Botani Putrajaya, Malaysia. Dried and ground stems were extracted with methanol for 72 h to give 25 g of crude extract. This was acidified with 5% hydrochloric acid, and non-alkaloidal material was removed followed with basification with 37% ammonium hydroxide to release the alkaloid. The alkaloid was extracted into chloroform to give 2.25 g of a crude alkaloid fraction. The fraction was dissolved in methanol and subjected to radial chromatography (4 mm thickness silica-gel plate) with dichloromethane:ethyl acetate followed by ethyl acetate:methanol with a gradual increase of solvent polarity. Rauniticine-*allo*-oxindole B was separated and purified by repeated preparative thin layer chromatography using chloroform:methanol (120:5). The polar fraction afforded colorless crystals when the solvent was allowed to evaporate (53 mg).

### Refinement

Carbon-bound H-atoms were placed in calculated positions [C—H 0.95 to 1.00 Å, *U*_iso_(H) 1.2 to 1.5*U*_eq_(C)] and were included in the refinement in the riding model approximation.

The oxygen-bound H-atoms were located in a difference Fourier map, and were refined with distance restraints of O–H 0.84±0.01 Å; their temperature factors were refined.

The absolute configuration was assumed to be that from a spectropic study (Seki *et al.*, 1993); in the absence of heavy atoms, 1853 Friedel pairs were merged.

### Crystal data

**Table:**

C_20_H_22_N_2_O_4_·CH_4_O	*F*(000) = 412
*M**_r_* = 386.44	*D*_x_ = 1.323 Mg m^−^^3^
Monoclinic, *P*2_1_	Mo *K*α radiation, λ = 0.71073 Å
Hall symbol: P 2yb	Cell parameters from 4552 reflections
*a* = 9.2330 (3) Å	θ = 2.4–29.2°
*b* = 7.2110 (2) Å	µ = 0.10 mm^−^^1^
*c* = 14.7678 (4) Å	*T* = 100 K
β = 99.313 (3)°	Prism, colorless
*V* = 970.27 (5) Å^3^	0.20 × 0.10 × 0.05 mm
*Z* = 2	

### Data collection

**Table:**

Agilent SuperNova Dual diffractometer with an Atlas detector	2381 independent reflections
Radiation source: SuperNova (Mo) X-ray Source	2181 reflections with *I* > 2σ(*I*)
Mirror	*R*_int_ = 0.039
Detector resolution: 10.4041 pixels mm^-1^	θ_max_ = 27.5°, θ_min_ = 2.4°
ω scans	*h* = −11→11
Absorption correction: multi-scan (*CrysAlis PRO*; Agilent, 2010)	*k* = −9→9
*T*_min_ = 0.981, *T*_max_ = 0.995	*l* = −19→19
9109 measured reflections	

### Refinement

**Table:**

Refinement on *F*^2^	Primary atom site location: structure-invariant direct methods
Least-squares matrix: full	Secondary atom site location: difference Fourier map
*R*[*F*^2^ > 2σ(*F*^2^)] = 0.037	Hydrogen site location: inferred from neighbouring sites
*wR*(*F*^2^) = 0.093	H atoms treated by a mixture of independent and constrained refinement
*S* = 1.05	*w* = 1/[σ^2^(*F*_o_^2^) + (0.0455*P*)^2^ + 0.2288*P*] where *P* = (*F*_o_^2^ + 2*F*_c_^2^)/3
2381 reflections	(Δ/σ)_max_ = 0.001
266 parameters	Δρ_max_ = 0.21 e Å^−^^3^
4 restraints	Δρ_min_ = −0.19 e Å^−^^3^

### Fractional atomic coordinates and isotropic or equivalent isotropic displacement parameters (Å^2^)

**Table:**

	*x*	*y*	*z*	*U*_iso_*/*U*_eq_	
O1	1.30437 (18)	1.0011 (3)	0.98971 (12)	0.0253 (4)	
O2	1.06782 (16)	0.9207 (3)	0.96906 (11)	0.0226 (4)	
O3	1.42440 (17)	0.5574 (3)	0.85208 (12)	0.0257 (4)	
O4	1.04085 (17)	0.6078 (3)	0.54863 (11)	0.0214 (4)	
O5	1.20768 (17)	1.2803 (3)	1.08162 (11)	0.0233 (4)	
N1	0.9975 (2)	0.3485 (3)	0.71523 (12)	0.0181 (4)	
N2	0.8390 (2)	0.7963 (3)	0.52546 (13)	0.0204 (4)	
C1	1.1933 (2)	0.8909 (3)	0.95551 (14)	0.0180 (5)	
C2	1.2329 (2)	0.7332 (4)	0.90209 (15)	0.0180 (5)	
C3	1.3739 (2)	0.6984 (4)	0.89692 (15)	0.0224 (5)	
H3	1.4447	0.7818	0.9280	0.027*	
C4	1.3173 (3)	0.4343 (4)	0.79913 (16)	0.0218 (5)	
H4	1.2837	0.4902	0.7373	0.026*	
C5	1.3986 (3)	0.2554 (4)	0.7878 (2)	0.0339 (6)	
H5A	1.4810	0.2807	0.7553	0.051*	
H5B	1.3316	0.1667	0.7525	0.051*	
H5C	1.4359	0.2032	0.8484	0.051*	
C6	1.1853 (2)	0.4113 (4)	0.84882 (15)	0.0188 (5)	
H6	1.2229	0.3627	0.9117	0.023*	
C9	1.0709 (2)	0.2749 (4)	0.80272 (15)	0.0200 (5)	
H9A	1.1188	0.1557	0.7924	0.024*	
H9B	0.9975	0.2512	0.8433	0.024*	
C10	0.9215 (2)	0.5222 (3)	0.72771 (15)	0.0163 (5)	
H10	0.8512	0.4990	0.7713	0.020*	
C11	1.0279 (2)	0.6712 (3)	0.76820 (14)	0.0168 (5)	
H11A	0.9735	0.7855	0.7786	0.020*	
H11B	1.0963	0.7006	0.7251	0.020*	
C12	1.1150 (2)	0.6013 (3)	0.86019 (15)	0.0164 (5)	
H12	1.0443	0.5862	0.9043	0.020*	
C13	0.8837 (3)	0.2265 (4)	0.66791 (16)	0.0229 (5)	
H13A	0.8237	0.1735	0.7114	0.028*	
H13B	0.9272	0.1239	0.6368	0.028*	
C14	0.7913 (3)	0.3531 (4)	0.59816 (15)	0.0201 (5)	
H14A	0.6853	0.3293	0.5968	0.024*	
H14B	0.8151	0.3324	0.5359	0.024*	
C15	0.8317 (2)	0.5561 (3)	0.63129 (15)	0.0167 (5)	
C16	0.9193 (2)	0.6518 (3)	0.56509 (15)	0.0179 (5)	
C17	0.7091 (2)	0.8212 (4)	0.56293 (15)	0.0189 (5)	
C18	0.6022 (2)	0.9545 (4)	0.54169 (16)	0.0223 (5)	
H18	0.6081	1.0452	0.4958	0.027*	
C19	0.4849 (3)	0.9504 (4)	0.59050 (16)	0.0242 (5)	
H19	0.4095	1.0408	0.5780	0.029*	
C20	0.4765 (2)	0.8163 (4)	0.65720 (16)	0.0239 (5)	
H20	0.3963	0.8172	0.6902	0.029*	
C21	0.5849 (2)	0.6802 (4)	0.67598 (15)	0.0205 (5)	
H21	0.5786	0.5875	0.7209	0.025*	
C22	0.7016 (2)	0.6830 (3)	0.62794 (14)	0.0165 (5)	
C23	1.2794 (3)	1.4465 (4)	1.11555 (17)	0.0266 (6)	
H23A	1.2064	1.5366	1.1290	0.040*	
H23B	1.3332	1.4979	1.0693	0.040*	
H23C	1.3483	1.4195	1.1718	0.040*	
H1	1.273 (3)	1.083 (4)	1.0219 (19)	0.041 (9)*	
H5	1.1180 (14)	1.304 (5)	1.065 (2)	0.043 (9)*	
H2	0.870 (3)	0.878 (3)	0.4885 (16)	0.034 (8)*	

### Atomic displacement parameters (Å^2^)

**Table:**

	*U*^11^	*U*^22^	*U*^33^	*U*^12^	*U*^13^	*U*^23^
O1	0.0214 (8)	0.0253 (10)	0.0282 (9)	−0.0040 (8)	0.0009 (7)	−0.0078 (8)
O2	0.0186 (8)	0.0237 (10)	0.0254 (8)	0.0002 (7)	0.0033 (6)	−0.0060 (8)
O3	0.0181 (8)	0.0299 (11)	0.0298 (9)	0.0030 (8)	0.0052 (7)	−0.0029 (8)
O4	0.0241 (8)	0.0194 (9)	0.0225 (8)	0.0002 (7)	0.0096 (6)	−0.0014 (7)
O5	0.0190 (8)	0.0246 (10)	0.0255 (9)	−0.0013 (8)	0.0017 (6)	−0.0026 (8)
N1	0.0226 (9)	0.0140 (10)	0.0174 (9)	0.0007 (8)	0.0022 (7)	−0.0008 (8)
N2	0.0237 (10)	0.0199 (11)	0.0180 (9)	−0.0018 (9)	0.0045 (8)	0.0034 (8)
C1	0.0185 (10)	0.0188 (13)	0.0155 (10)	−0.0008 (10)	−0.0004 (8)	0.0021 (9)
C2	0.0176 (10)	0.0196 (12)	0.0169 (10)	−0.0003 (9)	0.0033 (8)	0.0034 (9)
C3	0.0199 (10)	0.0266 (14)	0.0205 (11)	−0.0017 (11)	0.0026 (9)	0.0017 (11)
C4	0.0219 (11)	0.0238 (13)	0.0206 (10)	0.0037 (10)	0.0063 (9)	−0.0016 (11)
C5	0.0315 (13)	0.0286 (15)	0.0446 (16)	0.0093 (12)	0.0153 (12)	0.0015 (13)
C6	0.0209 (11)	0.0187 (12)	0.0169 (10)	0.0030 (10)	0.0027 (8)	0.0011 (10)
C9	0.0232 (11)	0.0160 (12)	0.0212 (11)	0.0008 (10)	0.0046 (9)	0.0035 (10)
C10	0.0182 (10)	0.0154 (11)	0.0159 (10)	0.0010 (9)	0.0051 (8)	0.0014 (9)
C11	0.0176 (10)	0.0153 (11)	0.0172 (10)	0.0013 (9)	0.0019 (8)	0.0001 (9)
C12	0.0173 (10)	0.0165 (11)	0.0156 (10)	0.0015 (9)	0.0035 (8)	0.0001 (9)
C13	0.0284 (12)	0.0188 (13)	0.0216 (11)	−0.0028 (10)	0.0040 (9)	−0.0010 (10)
C14	0.0241 (11)	0.0173 (12)	0.0191 (11)	−0.0031 (10)	0.0040 (9)	−0.0011 (10)
C15	0.0187 (10)	0.0157 (12)	0.0161 (10)	0.0002 (9)	0.0042 (8)	−0.0015 (9)
C16	0.0214 (10)	0.0173 (12)	0.0146 (10)	−0.0041 (9)	0.0015 (8)	−0.0036 (9)
C17	0.0198 (10)	0.0190 (13)	0.0168 (10)	−0.0048 (10)	−0.0001 (8)	−0.0024 (9)
C18	0.0258 (11)	0.0188 (12)	0.0198 (11)	−0.0026 (10)	−0.0039 (9)	0.0001 (10)
C19	0.0239 (11)	0.0209 (13)	0.0248 (12)	0.0033 (11)	−0.0052 (9)	−0.0036 (11)
C20	0.0209 (11)	0.0275 (15)	0.0234 (12)	0.0021 (11)	0.0033 (9)	−0.0040 (11)
C21	0.0226 (11)	0.0197 (12)	0.0186 (10)	−0.0016 (10)	0.0019 (9)	−0.0005 (10)
C22	0.0191 (10)	0.0140 (11)	0.0150 (10)	−0.0028 (9)	−0.0010 (8)	−0.0032 (9)
C23	0.0237 (11)	0.0261 (14)	0.0296 (13)	−0.0006 (11)	0.0030 (10)	−0.0057 (12)

### Geometric parameters (Å, °)

**Table:**

O1—C1	1.331 (3)	C10—C11	1.512 (3)
O1—H1	0.84 (3)	C10—C15	1.547 (3)
O2—C1	1.227 (3)	C10—H10	1.0000
O3—C3	1.338 (3)	C11—C12	1.547 (3)
O3—C4	1.458 (3)	C11—H11A	0.9900
O4—C16	1.228 (3)	C11—H11B	0.9900
O5—C23	1.421 (3)	C12—H12	1.0000
O5—H5	0.84 (3)	C13—C14	1.529 (3)
N1—C9	1.458 (3)	C13—H13A	0.9900
N1—C13	1.459 (3)	C13—H13B	0.9900
N1—C10	1.462 (3)	C14—C15	1.569 (3)
N2—C16	1.356 (3)	C14—H14A	0.9900
N2—C17	1.411 (3)	C14—H14B	0.9900
N2—H2	0.88 (3)	C15—C22	1.505 (3)
C1—C2	1.464 (3)	C15—C16	1.530 (3)
C2—C3	1.340 (3)	C17—C18	1.377 (3)
C2—C12	1.502 (3)	C17—C22	1.393 (3)
C3—H3	0.9500	C18—C19	1.395 (3)
C4—C5	1.515 (4)	C18—H18	0.9500
C4—C6	1.530 (3)	C19—C20	1.392 (4)
C4—H4	1.0000	C19—H19	0.9500
C5—H5A	0.9800	C20—C21	1.397 (3)
C5—H5B	0.9800	C20—H20	0.9500
C5—H5C	0.9800	C21—C22	1.383 (3)
C6—C9	1.522 (3)	C21—H21	0.9500
C6—C12	1.537 (3)	C23—H23A	0.9800
C6—H6	1.0000	C23—H23B	0.9800
C9—H9A	0.9900	C23—H23C	0.9800
C9—H9B	0.9900		
			
C1—O1—H1	109 (2)	H11A—C11—H11B	108.3
C3—O3—C4	117.88 (17)	C2—C12—C6	108.58 (17)
C23—O5—H5	108 (3)	C2—C12—C11	113.2 (2)
C9—N1—C13	113.6 (2)	C6—C12—C11	111.30 (19)
C9—N1—C10	111.33 (18)	C2—C12—H12	107.8
C13—N1—C10	104.68 (17)	C6—C12—H12	107.8
C16—N2—C17	111.6 (2)	C11—C12—H12	107.8
C16—N2—H2	125 (2)	N1—C13—C14	104.1 (2)
C17—N2—H2	122 (2)	N1—C13—H13A	110.9
O2—C1—O1	121.6 (2)	C14—C13—H13A	110.9
O2—C1—C2	123.3 (2)	N1—C13—H13B	110.9
O1—C1—C2	115.10 (19)	C14—C13—H13B	110.9
C3—C2—C1	120.4 (2)	H13A—C13—H13B	109.0
C3—C2—C12	120.3 (2)	C13—C14—C15	105.55 (18)
C1—C2—C12	119.10 (19)	C13—C14—H14A	110.6
O3—C3—C2	126.2 (2)	C15—C14—H14A	110.6
O3—C3—H3	116.9	C13—C14—H14B	110.6
C2—C3—H3	116.9	C15—C14—H14B	110.6
O3—C4—C5	105.80 (19)	H14A—C14—H14B	108.8
O3—C4—C6	109.42 (18)	C22—C15—C16	101.90 (19)
C5—C4—C6	114.1 (2)	C22—C15—C10	115.61 (18)
O3—C4—H4	109.1	C16—C15—C10	113.55 (18)
C5—C4—H4	109.1	C22—C15—C14	114.13 (18)
C6—C4—H4	109.1	C16—C15—C14	110.19 (18)
C4—C5—H5A	109.5	C10—C15—C14	101.82 (18)
C4—C5—H5B	109.5	O4—C16—N2	124.5 (2)
H5A—C5—H5B	109.5	O4—C16—C15	127.2 (2)
C4—C5—H5C	109.5	N2—C16—C15	108.30 (19)
H5A—C5—H5C	109.5	C18—C17—C22	122.9 (2)
H5B—C5—H5C	109.5	C18—C17—N2	128.5 (2)
C9—C6—C4	113.91 (19)	C22—C17—N2	108.6 (2)
C9—C6—C12	110.48 (18)	C17—C18—C19	117.1 (2)
C4—C6—C12	109.8 (2)	C17—C18—H18	121.5
C9—C6—H6	107.5	C19—C18—H18	121.5
C4—C6—H6	107.5	C20—C19—C18	121.2 (2)
C12—C6—H6	107.5	C20—C19—H19	119.4
N1—C9—C6	110.5 (2)	C18—C19—H19	119.4
N1—C9—H9A	109.5	C19—C20—C21	120.5 (2)
C6—C9—H9A	109.5	C19—C20—H20	119.7
N1—C9—H9B	109.5	C21—C20—H20	119.7
C6—C9—H9B	109.5	C22—C21—C20	118.7 (2)
H9A—C9—H9B	108.1	C22—C21—H21	120.6
N1—C10—C11	111.33 (17)	C20—C21—H21	120.6
N1—C10—C15	102.46 (18)	C21—C22—C17	119.6 (2)
C11—C10—C15	117.98 (19)	C21—C22—C15	131.2 (2)
N1—C10—H10	108.2	C17—C22—C15	109.2 (2)
C11—C10—H10	108.2	O5—C23—H23A	109.5
C15—C10—H10	108.2	O5—C23—H23B	109.5
C10—C11—C12	109.15 (19)	H23A—C23—H23B	109.5
C10—C11—H11A	109.8	O5—C23—H23C	109.5
C12—C11—H11A	109.8	H23A—C23—H23C	109.5
C10—C11—H11B	109.8	H23B—C23—H23C	109.5
C12—C11—H11B	109.8		
			
O2—C1—C2—C3	−173.7 (2)	N1—C10—C15—C22	158.78 (18)
O1—C1—C2—C3	5.8 (3)	C11—C10—C15—C22	−78.6 (3)
O2—C1—C2—C12	0.8 (3)	N1—C10—C15—C16	−83.9 (2)
O1—C1—C2—C12	−179.7 (2)	C11—C10—C15—C16	38.7 (3)
C4—O3—C3—C2	3.7 (4)	N1—C10—C15—C14	34.5 (2)
C1—C2—C3—O3	178.6 (2)	C11—C10—C15—C14	157.11 (19)
C12—C2—C3—O3	4.1 (4)	C13—C14—C15—C22	−135.9 (2)
C3—O3—C4—C5	−158.8 (2)	C13—C14—C15—C16	110.2 (2)
C3—O3—C4—C6	−35.5 (3)	C13—C14—C15—C10	−10.6 (2)
O3—C4—C6—C9	−176.11 (19)	C17—N2—C16—O4	−175.5 (2)
C5—C4—C6—C9	−57.8 (3)	C17—N2—C16—C15	5.0 (2)
O3—C4—C6—C12	59.4 (2)	C22—C15—C16—O4	174.9 (2)
C5—C4—C6—C12	177.6 (2)	C10—C15—C16—O4	49.9 (3)
C13—N1—C9—C6	−178.73 (19)	C14—C15—C16—O4	−63.6 (3)
C10—N1—C9—C6	−60.9 (2)	C22—C15—C16—N2	−5.7 (2)
C4—C6—C9—N1	−68.4 (3)	C10—C15—C16—N2	−130.7 (2)
C12—C6—C9—N1	55.7 (2)	C14—C15—C16—N2	115.8 (2)
C9—N1—C10—C11	62.3 (2)	C16—N2—C17—C18	178.7 (2)
C13—N1—C10—C11	−174.57 (18)	C16—N2—C17—C22	−2.0 (3)
C9—N1—C10—C15	−170.65 (18)	C22—C17—C18—C19	1.9 (3)
C13—N1—C10—C15	−47.5 (2)	N2—C17—C18—C19	−178.8 (2)
N1—C10—C11—C12	−57.0 (2)	C17—C18—C19—C20	−0.4 (3)
C15—C10—C11—C12	−174.97 (18)	C18—C19—C20—C21	−0.9 (4)
C3—C2—C12—C6	20.8 (3)	C19—C20—C21—C22	0.9 (3)
C1—C2—C12—C6	−153.8 (2)	C20—C21—C22—C17	0.5 (3)
C3—C2—C12—C11	−103.4 (3)	C20—C21—C22—C15	−178.8 (2)
C1—C2—C12—C11	82.0 (2)	C18—C17—C22—C21	−2.0 (3)
C9—C6—C12—C2	−177.57 (17)	N2—C17—C22—C21	178.6 (2)
C4—C6—C12—C2	−51.1 (2)	C18—C17—C22—C15	177.5 (2)
C9—C6—C12—C11	−52.3 (2)	N2—C17—C22—C15	−1.9 (2)
C4—C6—C12—C11	74.2 (2)	C16—C15—C22—C21	−176.1 (2)
C10—C11—C12—C2	175.10 (18)	C10—C15—C22—C21	−52.5 (3)
C10—C11—C12—C6	52.4 (2)	C14—C15—C22—C21	65.2 (3)
C9—N1—C13—C14	162.01 (19)	C16—C15—C22—C17	4.5 (2)
C10—N1—C13—C14	40.4 (2)	C10—C15—C22—C17	128.1 (2)
N1—C13—C14—C15	−17.1 (2)	C14—C15—C22—C17	−114.2 (2)

### Hydrogen-bond geometry (Å, °)

**Table:**

*D*—H···*A*	*D*—H	H···*A*	*D*···*A*	*D*—H···*A*
O1—H1···O5	0.84 (3)	1.83 (3)	2.662 (3)	173 (4)
O5—H5···O2^i^	0.84 (3)	1.91 (3)	2.728 (2)	165 (4)
N2—H2···O4^ii^	0.88 (3)	1.97 (3)	2.805 (3)	158 (3)

Symmetry codes: (i) −*x*+2, *y*+1/2, −*z*+2; (ii) −*x*+2, *y*+1/2, −*z*+1.
